# Opportunistic Hybrid Transport Protocol (OHTP) for Cognitive Radio Ad Hoc Sensor Networks

**DOI:** 10.3390/s151229871

**Published:** 2015-12-15

**Authors:** Yousaf Bin Zikria, Summera Nosheen, Farruh Ishmanov, Sung Won Kim

**Affiliations:** 1 Department of Information and Communication Engineering, Yeungnam University, 280, Daehak-Ro, Gyeongsan, Geongbuk 38541, Korea; yousafbinzikria@ynu.ac.kr; 2 Comsats Institute of Information Technology, Park Rd, Islamabad 45550, Pakistan; sumera_16@hotmail.com; 3 Department of Electronics and Communication Engineering, Kwangwoon University, 447-1 Wolgye-dong, Nowon-gu, Seoul 01897, Korea; farruh.uzb@gmail.com

**Keywords:** transport protocol, cognitive radio ad hoc sensor network, opportunistic, congestion control

## Abstract

The inefficient assignment of spectrum for different communications purposes, plus technology enhancements and ever-increasing usage of wireless technology is causing spectrum scarcity. To address this issue, one of the proposed solutions in the literature is to access the spectrum dynamically or opportunistically. Therefore, the concept of cognitive radio appeared, which opens up a new research paradigm. There is extensive research on the physical, medium access control and network layers. The impact of the transport layer on the performance of cognitive radio ad hoc sensor networks is still unknown/unexplored. The Internet’s *de facto* transport protocol is not well suited to wireless networks because of its congestion control mechanism. We propose an opportunistic hybrid transport protocol for cognitive radio ad hoc sensor networks. We developed a new congestion control mechanism to differentiate true congestion from interruption loss. After such detection and differentiation, we propose methods to handle them opportunistically. There are several benefits to window- and rate-based protocols. To exploit the benefits of both in order to enhance overall system performance, we propose a hybrid transport protocol. We empirically calculate the optimal threshold value to switch between window- and rate-based mechanisms. We then compare our proposed transport protocol to Transmission Control Protocol (TCP)-friendly rate control, TCP-friendly rate control for cognitive radio, and TCP-friendly window-based control. We ran an extensive set of simulations in Network Simulator 2. The results indicate that the proposed transport protocol performs better than all the others.

## 1. Introduction

Extensive enhancements in wireless technologies and ever-increasing bandwidth-intensive applications require more spectrum resources. The current spectrum allocation policy gives full rights only to licensed operators. However, this is one of the factors contributing to spectrum underutilization. A Federal Communications Commission (FCC) survey identified the inefficient usage of assigned spectrum [1]. To solve this issue, the cognitive radio network (CRN) [2] was introduced to exploit the holes in spectrum bands. A CRN is a viable solution to improve the efficiency of spectrum usage and network capacity. There are two types of users in a CRN: the licensed, or primary, users (PUs) and the unlicensed, or secondary, users (SUs). The SUs opportunistically access the spectrum band when the PUs are not using it. In a CRN, the primary constraint is to protect PU transmissions. Therefore, when a PU arrives, any SU must vacate the channel immediately. 

The CRN may consist of a single channel or be multi-channel. With a single channel, SUs have to wait for availability of the channel for communications. In multi-channel scenarios, the SUs still have to leave a channel upon PU arrival. However, if another channel is available, they can switch to that channel and resume communications. Otherwise, SUs wait until another channel is free. 

The cognitive radio ad hoc network (CRAHN) [3] does not have any infrastructure backbone. Cognitive radio (CR) users communicate with other CR users in an ad hoc manner on both licensed and unlicensed spectrum bands. In a CRAHN, each user needs to have all the CR capabilities and is responsible for all communications actions based on local observation. 

The cognitive radio ad hoc sensor network (CRASN) [4] is a specialized ad hoc network of distributed wireless sensors that are equipped with cognitive radio capabilities. A wireless node selects a vacant channel for communications and leaves the channel upon the arrival of a licensed user. Cognitive radio is one of the most promising techniques to enhance the efficiency of wireless sensor networks (WSNs). A CRASN increases spectrum utilization, increases network efficiency, and extends the lifetime of the WSN [5]. 

In this paper, we propose a new transport protocol named opportunistic hybrid transport protocol for cognitive radio ad hoc sensor networks (OHTP-CRASN). The research objective is to provide a new congestion control mechanism by considering the dynamic nature of cognitive radio ad hoc sensor networks. Furthermore, it takes advantage of window-based and rate-based protocols according to the network conditions to improve overall system performance. 

The rest of the paper is organized as follows. Section 2 discusses the related work. In Section 3, we explain our proposed opportunistic hybrid transport protocol for cognitive radio ad hoc sensor networks. Section 4 provides the details of the simulation environment and discusses the results. Finally, Section 5 concludes the paper.

## 2. Related Work

Transmission Control Protocol (TCP) [6] is the *de facto* transport layer connection-oriented and reliable end-to-end protocol. It provides reliable data delivery with the help of flow control and congestion control mechanisms. TCP is the window-based protocol that uses sequence numbers and acknowledgements (ACKs) to attain reliability. The sender and receiver exchange data associated with segments. When the receiver receives a segment, it sends an acknowledgement to the sender indicating correct reception of the segment. On successful reception of an ACK, the sender transmits the next segment. If the sender gets two ACKs for the same segment, it is called a duplicate ACK. On reception of three duplicate ACKs, the sender assumes that segment and its data were lost and retransmits. Furthermore, TCP also employs a timeout mechanism to detect losses. It starts a timer immediately after transmitting a segment and waits for the timeout event. If it gets an ACK before the timer expires, it considers the data successfully delivered. Otherwise, on the occurrence of a timeout, it considers the segment and its data as lost and retransmits the same segment. The timeout event forces TCP to initiate the slow start algorithm. 

**Figure 1:**
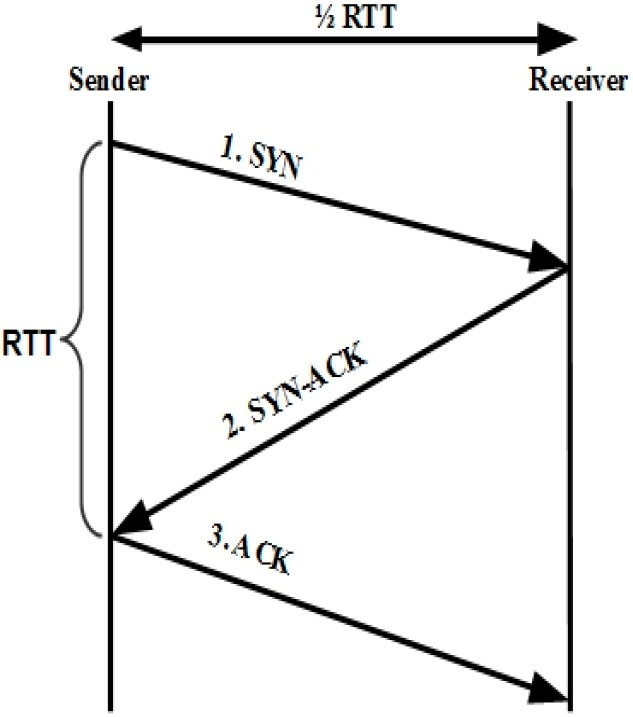
TCP RTT sample calculation.

The timeout interval is called retransmission timeout (RTO) [7]. TCP adjusts its window according to the network conditions by using an additive-increase multiplicative-decrease (AIMD) [6,8] strategy. TCP starts the window with one packet or some large value. Moreover, the window is increased exponentially by one segment for every successive ACK until the source reaches network capacity. This phase is called slow start (ss), and the capacity estimate is called the ss threshold (ssthresh). Hereafter, congestion avoidance (CA) starts, and the window is increased by one segment for every round-trip time (RTT) until loss is detected. There are two methods to detect packet loss, as explained above: RTO expiration and three duplicate ACKs. In the case of loss, TCP considers the cause to be network congestion and reduces the current congestion window to half. Network congestion means that the receiver’s network resources are overloaded due to the current sender rate. Hence, packet loss and delay increase, and the network can collapse in the worst-case scenario. To cope with this critical issue, many variants of TCP, like Tahoe [6], Reno [9], New Reno [10], SACK [11], Vegas [12], *etc.*, offer different solutions to handle congestion control and avoidance issues. TCP estimates round-trip time using the SYN estimator. The sender sends the SYN segment to the receiver, and the receiver sends back a SYN-ACK on successfully receiving the segment. Then, the sender responds to the SYN-ACK segment with an ACK segment. The time interval between the arrival of a SYN segment and the arrival of an ACK segment is called the RTT of the connection. RTT indicates the minimum total transmission and propagation delay. The RTT estimator is illustrated in Figure 1.

TCP CRAHN [13] is the variant of TCP and based on the classical TCP NewReno. They added the functionality of channel switching and periodic switching. However, TCP CRAHN congestion control is completely the same as TCP. The functionality of periodic sensing is achieved using the extra message to the nodes in the routing path. This approach is not scalable as the nodes have to accommodate multiple flows from different TCP sources. Due to the additional states and control messages, it is difficult to implement and requires additional buffer space along the path. Moreover, it requires feedback information from relay nodes and cross-layer collaboration from lower layers. Hence, the end-to-end principle is broken [14].

RETP [15] is proposed to prolong the lifetime of nodes by energy efficiency. Every node determines its operating channel in a distributed manner although the scheduling of sending data is determined centrally by a sink node. They assumed that every node can reach the sink in one hop. Hence, it is not scalable and cannot be used in multihop environment. 

In contrast to window-based congestion mechanisms, rate-based control was proposed to address congestion handling under TCP. TCP-friendly rate control (TFRC) [16] is the first rate-based transport protocol. These authors proposed increasing the sending rate slowly in response to a decrease in the loss event rate. It does not halve the sending rate in response to a single loss event. However, in the case of several consecutive losses, it halves the sending rate. To adjust the sending rate, the receiver informs the sender as to the reception of packets per round-trip time. The sender will reduce the sending rate if it has not received feedback for consecutive round-trip times. The loss event rate is calculated at the receiver, and the receiver provides it to the sender. Thereafter, the sender will adjust the sending rate accordingly. It uses the method called Average Loss Interval (ALI) to obtain the smoothed sending rate. This smoothing is done in order to circumvent violent reaction to a single unexpected loss event. 

**Figure 2:**
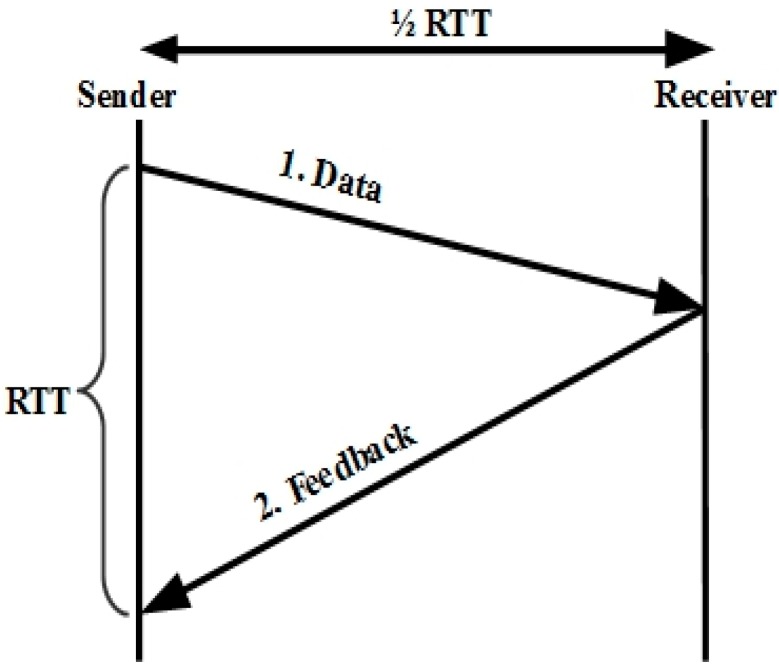
TFRC RTT sample calculation.

There are three main factors determining TFRC throughput: TCP-friendly equations, loss event-rate estimation, and network delays. The sender calculates a new RTT sample whenever a feedback packet is received. Figure 2 illustrates the TFRC RTT sample calculation. When a feedback packet is received, the sender performs the following five steps: (i) calculate the most recent sample of the RTT; (ii) estimate a new smoothed RTT; (iii) calculate the TCP retransmission timeout value; (iv) adjust the sending rate; and (v) reset the non-feedback timer. 

TFRC ensures a smooth transmit rate using the following function:

(1) $$T=\;\frac{s}{R+\sqrt{\frac{2p}{3}}+\;t_{RTO}.\left(3\sqrt{\frac{3p}{8}}\right).p.\left(1+32p^{2}\right)}$$

**Figure 3:**
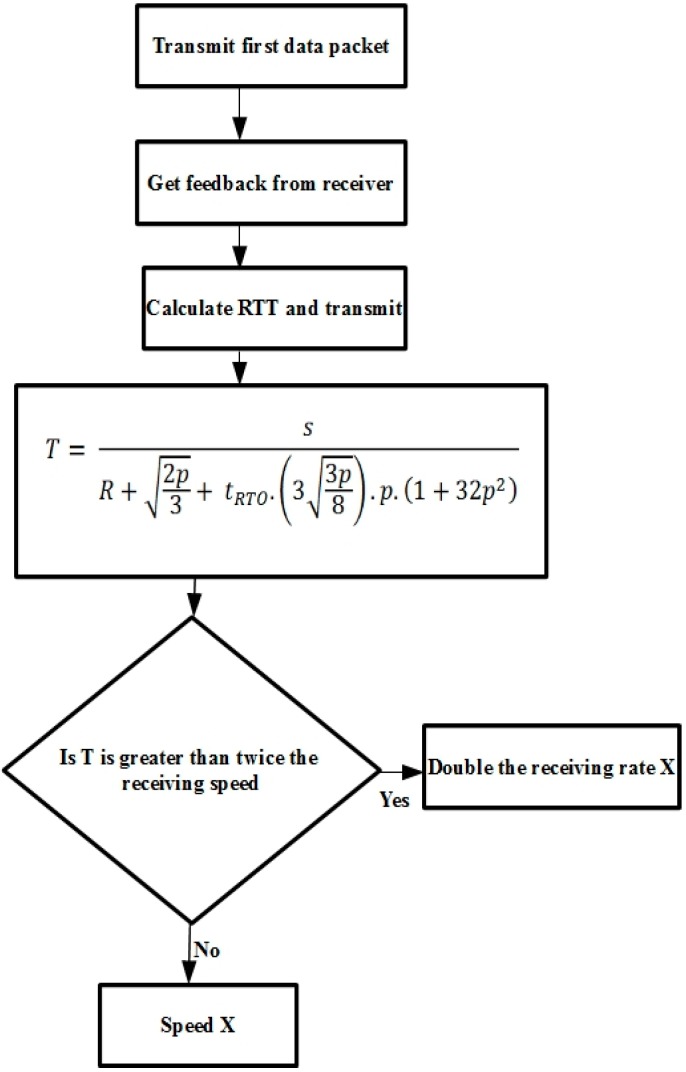
TFRC congestion control mechanism.

Sending rate *T* is a function of the packet size, the round-trip time *R*, the TCP retransmit timeout *t_RTO_*, and loss event rate *p*. Sender and receiver both have to measure the packet size, round-trip time, and loss event rate. *t_RTO_* is set to 4 × *R* for approximation, and this is reasonable for acquiring TCP friendliness [17]. Figure 3 shows the TFRC congestion control mechanism.

In order to utilize bandwidth more efficiently, TCP-Friendly Rate Control for Cognitive Radio (TFRC-CR) [18] was proposed. It allows instant changes in the sender rate based on spectrum-related changes. TFRC-CR adjusts the sending rate by identifying and differentiating between spectrum change and true congestion in the network. Furthermore, it provides the time to restart transmissions after a pause upon PU arrival. TFRC-CR enhances overall TFRC and fits into the CRN paradigm. Despite this, TFRC-CR responds slowly to an immediate decrease in the loss event rate. This is due to a flaw in the computation of the loss event rate that relies on loss event history. Additionally, in the case of a spectrum change to an idle spectrum with larger capacity, it takes a longer time to adjust the rate to the newly available capacity. Thus, it affects RTT and RTO values.

Some researchers [19,20] suggested sender-based measurement of RTT because most end users lack processing capabilities. In addition, TFRC lacks the fine-grained congestion avoidance mechanism that TCP ACK-clocking provides. Therefore, a new mechanism called TCP-friendly window-based control (TFWC) [21] was proposed. It modifies the TFRC throughput model according to Equation (2) to fit a window-based mechanism.

(2) $$W=\;\frac{1}{\sqrt{\frac{2p}{3}}+\;\left(12\sqrt{\frac{3p}{8}}\right).p.\left(1+32p^{2}\right)}$$

where *W* is the window size in the packets, and p is the loss event rate. The sender computes the packet loss and calculates the congestion window (*cwnd*) using the TFWC equation. If the sequence number of the new data waiting to be sent fulfills Equation (3), the ACK-clock is generated.

(3) **sequence number of new data** ≤ ***cwnd*** + **unacknowleged sequence number**

**Figure 4:**
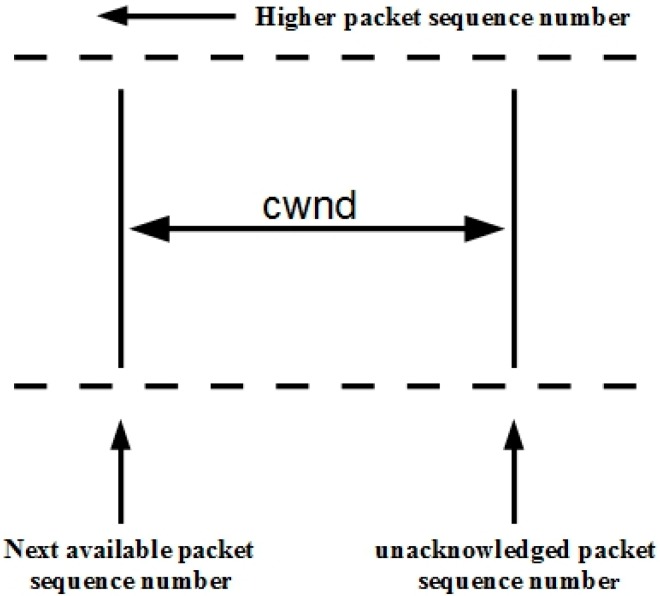
TFWC congestion window mechanism.

The TFWC *cwnd* mechanism is presented in Figure 4. The TFWC receiver provides an acknowledgement vector (AckVec) and time-stamp echo to the sender to calculate the *cwnd* and RTT. If the ACK flow is disrupted, the TFWC smoothness is reduced. This protocol is proposed for wired networks, and therefore, cannot handle the challenges related to a CRN, such as frequent spectrum changes due to PU activity. Hence, the losses due to the dynamic nature of the network are considered congestion. As a result, it adversely affects the sending rate and underutilizes network capacity.

## 3. OHTP-CRASN

The paradigm of the transport protocol changes in a CRASN and needs to adapt to the dynamic nature of the CRN. The first and foremost important thing is to detect the PU during transmissions. Afterwards, losses during the handover from one channel to a vacant channel should not be considered congestion. In fact, these losses are interruption losses [22]. If it is not tackled carefully, the performance of the transport protocol is greatly hampered. Hence, in an efficient transport protocol, differentiating between fake and true congestion is essential. We use the FCC-specified database and connectivity directions [23] to detect the PU appearance at a specific instant in time on a tagged channel. The advantage of FCC-mandated databases is that it allows the transport protocol to integrate with a designated spectrum database. It contains information about PU’s location, channel, transmission power and activity over time. There is no need for the feedback from the intermediate nodes or underlying layers. The database is polled only when it is needed. Whenever the packet loss is deteced, it immediately checks the PU’s activity. We consider it opportunistic because all the channels are homogeneous, and shifting to the new channel while retaining the same sending rate may provide an opportunity to deliver more data in a given time. Hence, exploring the window of opportunity will enhance system throughput.

**Figure 5:**
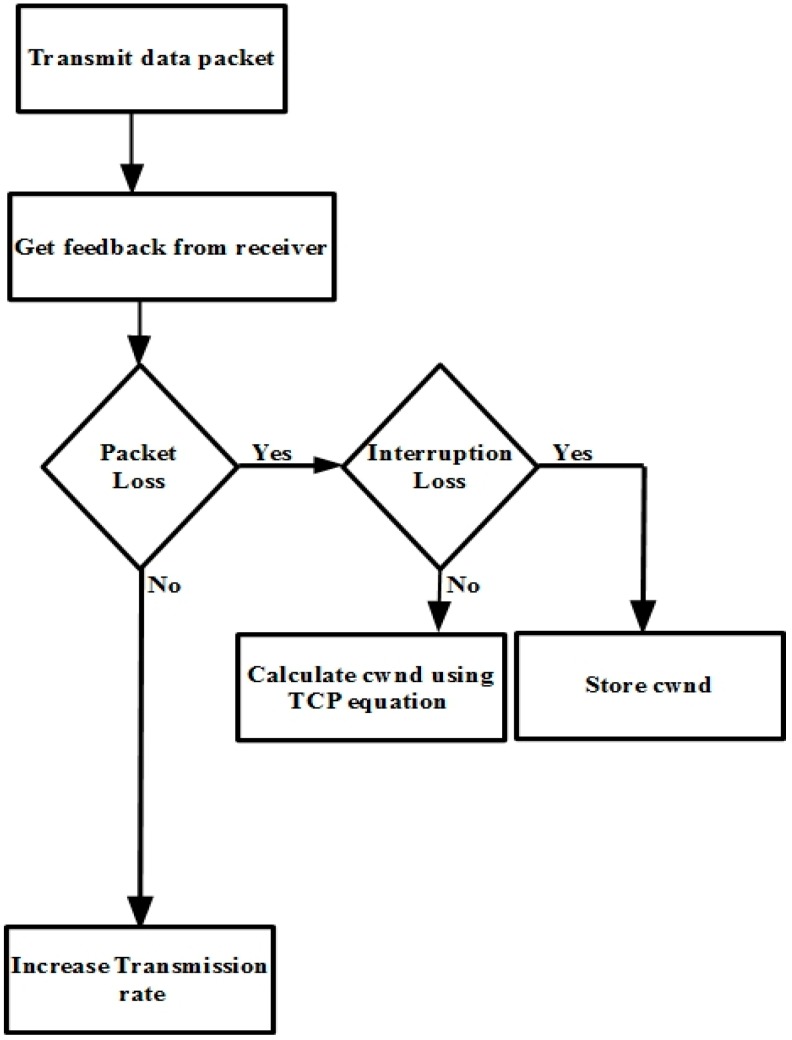
OHTP congestion control mechanism.

Figure 5 portrays OHTP congestion control mechanism. OHTP is the sender-driven congestion control protocol. It calculates the transmission rate with the help of receiver feedback. The receiver reports feedback at least once per round-trip time. The feedback contains the number of packets received by receiver. The source calculates *cwnd* upon every feedback. In the case of packet loss, the sender immediately checks for any PU activity on specific channel using the FCC-specified database. If PU is active, it reaffirms the interruption loss and stores the *cwnd*. After moving to the next available channel, it resumes the rate from the previous channel. In case the loss is due to erroneous channel, it determines the *cwnd* using TCP throughput equation. In addition, it reduces the *cwnd* by packet loss ratio. In the event of no packet loss, transmission rate increases until the upper limit is reached.

**Figure 6:**
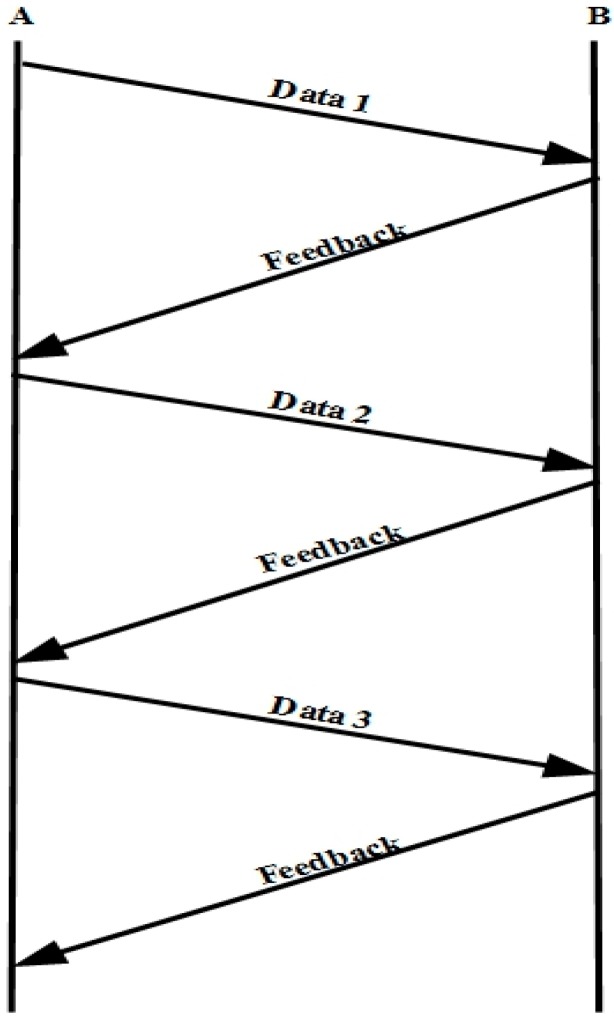
OHTP timing diagram.

**Figure 7:**
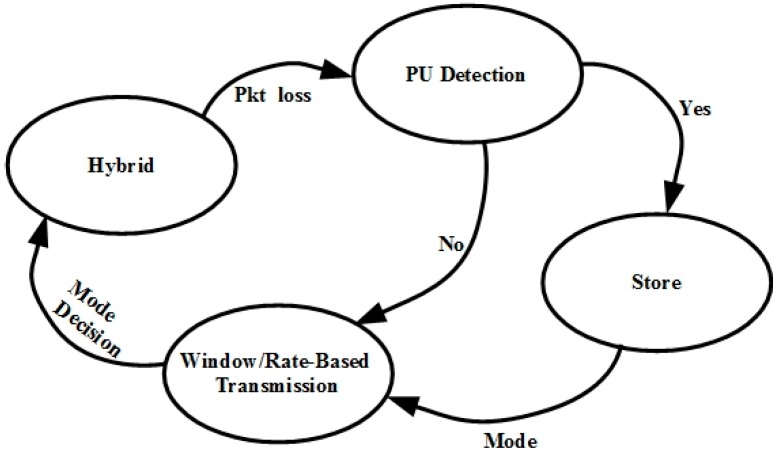
OHTP FSM.

Figure 6 depicts the OHTP timing diagram. Here, the data represent the packets transmitted and feedback means the list of packets successfully received by receiver. By checking the feedback, the sender knows the lost packets. The sender looks into the feedback for any missing packets excluding the last three packets by applying TCP three duplicate rule. When the packet loss is detected, it determines whether it is the interruption loss or the normal loss. If it is the interruption loss, it keeps the current *cwnd* for further transmission. Otherwise, it calculates the *cwnd* using the TCP throughput equation and additionally reduces the *cwnd* by packet loss ratio. The OHTP finite state machine (FSM) is shown in Figure 7.

### 3.1. Hybrid State

This is the default state of the OHTP-CRASN. The protocol returns to this state after interruption. The protocol starts with a linear increase of *cwnd* until a packet loss event occurs. On the occurrence of packet loss, it instantly enters the PU detection state. This state is also responsible for calculating the sending rate for packets in bytes per second based on the decision from the window/rate-based state. In the case of packet loss due to fading or an erroneous channel, the transmission rate is directly calculated with the help of a TCP throughput equation. We adjust the transmission rate in bytes or packets by looking at the loss interval history. If there is 10% packet loss, then we reduce the window by the same ratio. However, packet loss due to PU appearance is not treated as channel congestion. The transmission rate is not changed. This is the opportunistic approach, which increases throughput aggressively.

### 3.2. Expected PU Detection State

This state immediately checks the database to verify whether there is any PU activity on the current channel or not. If the database confirms PU activity, it moves to the store state. Otherwise, it goes to the window/rate-based state.

### 3.3. Store State

In this state, the packet loss is due to PU activity and indicates interruption loss. Hence, it keeps the current values of *cwnd* and the sending rate for further transmission. It is the responsibility of the underlying link layer algorithm to find an alternate vacant channel for transmission or to pause the transmission.

### 3.4. Window/Rate-Based Transmission State

This state is specifically designed to choose either window- or rate-based transmission by looking at *cwnd* size. According to the empirical results obtained in our simulation, rate-based transmission works well for low *cwnd* values, and window-based transmission is a better option for higher *cwnd* values. To achieve the highest throughput and lesser delay, we set 70 as the optimal threshold value from the simulation results presented in the next section. Rate-based transmission is active when *cwnd* is below 70, and window-based transmission is active at other times.

The proposed and compared protocols are designed for interactive streaming applications. It is needless to retransmit the video/voice packet considering the long delay for retransmission. Further, the two common reasons that inhibit the use of TCP for real-time interactive streaming services are Additive-Increase Multiplicative-Decrease (AIMD) and retransmission. In summary, the interactive multimedia applications require the timely packet delivery and smooth predictable transmission rate.

## 4. Simulation Model and Analysis

We conducted an extensive set of simulations in Network Simulator 2 (ns2) [24]. In all the experimental studies, the simulation parameters are the same to provide a fair comparison. As explained earlier, upon the arrival of a PU, the SU has to immediately vacate the channel and look for a vacant channel to resume communications. Otherwise, the SU pauses communications until the PU transmission ends. It is very important to investigate the overall impact of all the transport protocols vigorously in the presence of PU activity. PU activity is independent and random during the simulation. However, it is the same for all the simulations to maintain fairness. The multi-channel CRASN contains 11 channels for communications. All the channels have homogeneous capacity. The parameters used in the simulations are given in Table 1.

**Table 1:** Simulation Parameters.

Radio Propagation Model	Two Ray Ground
Channel Type	Wireless Channel
Network Interface Type	Wireless Phy
Mac Type	802.11
Antenna Model	Omni Antenna
Transport Layer	TFRC-CR/TFRC/TFWC/OHTP-CRASN
Queue Length	100
Cognitive Radio Model	CRAHN
Simulation Time	500 s
Simulation Area	1000 m × 1000 m

**Figure 8:**
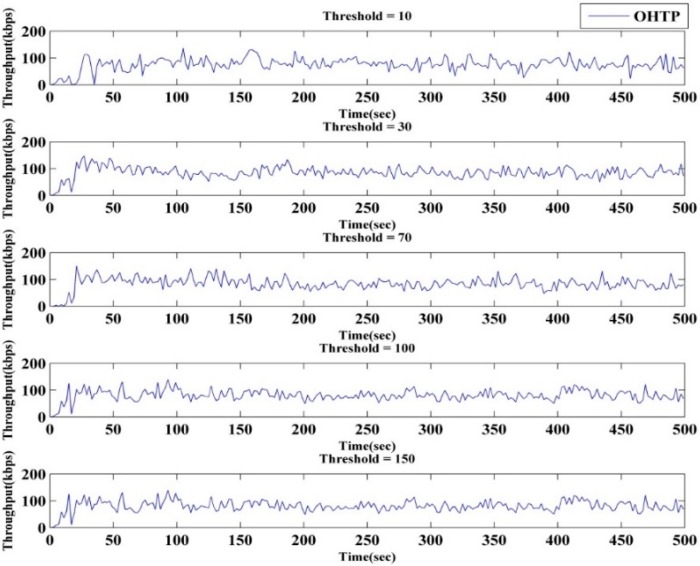
Optimum threshold value.

**Figure 9:**
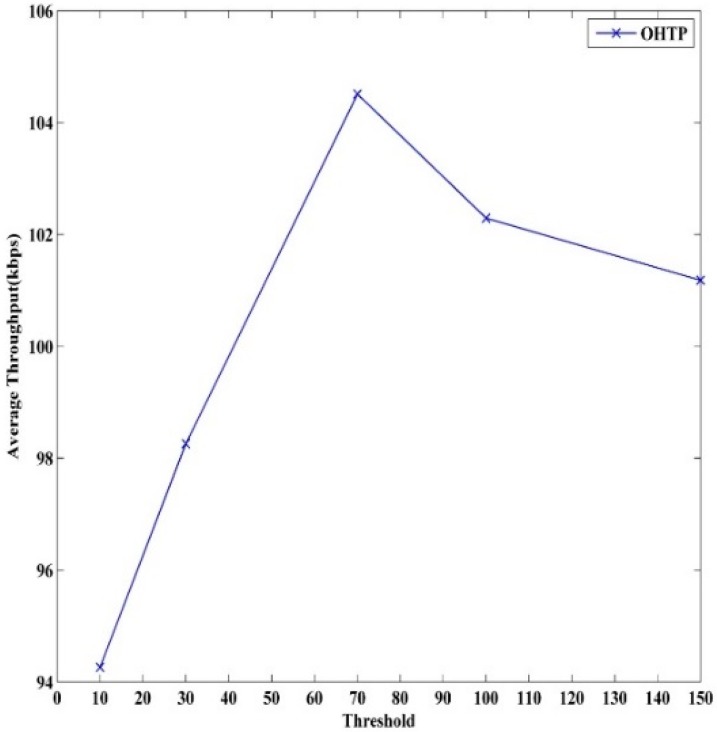
Threshold *versus* average throughput.

OHTP-CRASN performance depends highly on the threshold value to choose window- or rate-based transmission. We find the optimal value with the help of the empirical results. Figure 8 shows time *versus* throughput for different threshold values. The results indicate that fewer data are delivered when the threshold value is lower. However, it shows improvement with an increasing threshold value. Throughput starts to deteriorate after 100. There is not much difference in the results obtained for threshold values of 100 and 70. Nevertheless, the latter threshold value depicts slightly less packet loss and delay. Figure 9 depicts average throughput against the threshold. Therefore, according to the simulation outcome, we set the optimum threshold value to 70.

**Figure 10:**
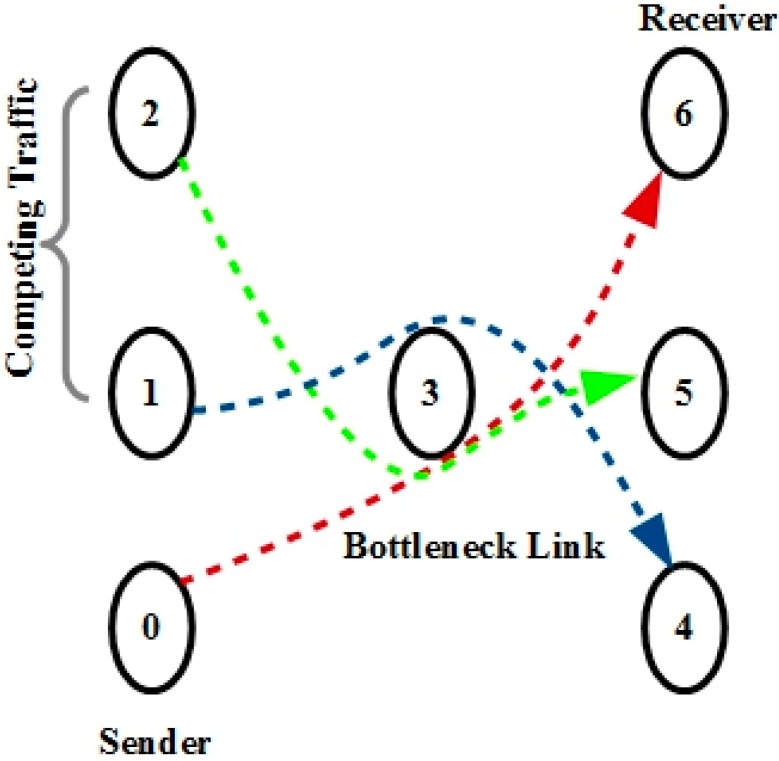
Dumbbell topology.

Assume the scenario of a CRASN in which a set of multimedia sensors has been attached with a covert battalion, which transmits multimedia to the sink. It generates bursty traffic in a multihop environment. On a given path, the link with the minimum capacity is called a narrow link, and the link with the minimum available bandwidth is called a tight link. When the link is both narrow and tight on a given path, it is called a bottleneck. The dumbbell topology [25,26] is the perfect example of this and is shown in Figure 10. In this topology, m flows are generated at the source nodes and directed towards sink nodes using the bottleneck link. All flows compete for the finite bandwidth. This realistic network scenario is designed to evaluate how the transport protocols perform under the pressure of other sensor nodes with competing traffic that are sharing the same link, and furthermore, how performance is affected by PU activity.

**Figure 11:**
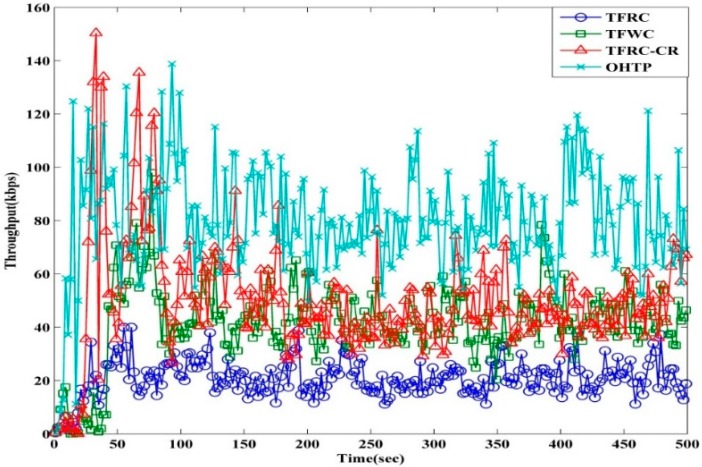
Instantaneous throughput of TFRC, TFWC, TFRC-CR, and OHTP.

Figure 11 depicts the instantaneous throughput of TFRC, TFWC, TFRC-CR, and OHTP. It can be seen from the figure that TFRC performs the worst among all the protocols. Even though it is designed to give a smoother transmission rate, it fails to identify true congestion. It is evident that the interruption caused by the PU and the resulting packet losses misleads TFRC to detect this as congestion. As a result, it reduces the transmission rate and eventually underutilizes the bandwidth. TFWC performs better than TFRC, but it also lacks information to identify PU activity and to adapt the transmission rate accordingly. TFWC relies on the loss history. This helps to calculate the average loss interval (ALI). The calculation of ALI impacts the next *cwnd*. On the occurrence of packet losses, it advances *cwnd* very slowly. Hence, it underutilizes the available capacity. TFRC-CR performs better than TFRC and TFWC, as can be clearly seen from the diagram. It can differentiate between true congestion and interruption losses. Therefore, it can handle and adapt according to PU activity. However, TFRC-CR reacts very slowly to the immediate decrease in the loss event rate. Subsequently, when transmission changes to an idle channel with available capacity, it will take a longer time to adjust the rate to the newly available capacity. This is clearly visible from the results presented in Figure 11. Our proposed OHTP performs the best among all the competing transport protocols. OHTP smartly detects packet loss due to PU activity and maintains the transmission rate to exploit the new available capacity. Hence, it achieves greater throughput. Moreover, it adjusts the transmission rate from true congestion accordingly. This results in an effective utilization of available spectrum and enhances overall system throughput.

Table 2 shows the percentage throughput improvement of OHTP over TFRC-CR, TFWC, and TFRC. OHTP is 284.19% superior to TFRC. Furthermore, OHTP performs 92.51% better than TFWC. Our proposed scheme, OHTP, achieves 58.4% higher throughput than TFRC-CR. Hence, the results reaffirm that our proposed OHTP is better than the other compared transport protocols.

**Table 2:** OHTP percentage throughput improvement over transport protocols.

TFRC	TFWC	TFRC-CR
284.19	92.51	58.4

**Figure 12:**
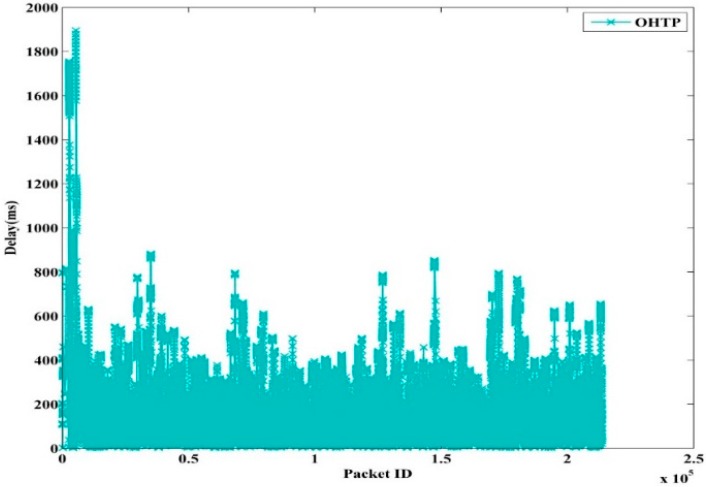
OHTP Packet Delay.

Figure 12 shows the instantaneous delay of OHTP. It is obvious that it incurs greater delay due to more waiting time in the queue. In addition, it is expected that high throughput is achieved at the cost of high delay [27]. The proposed scheme efficiently maintains the transmission rate under PU interruption. Therefore, PU arrival and shifting to the new channel do not have much affect on OHTP. As a result, the successful reception of data takes a longer time. 

**Figure 13:**
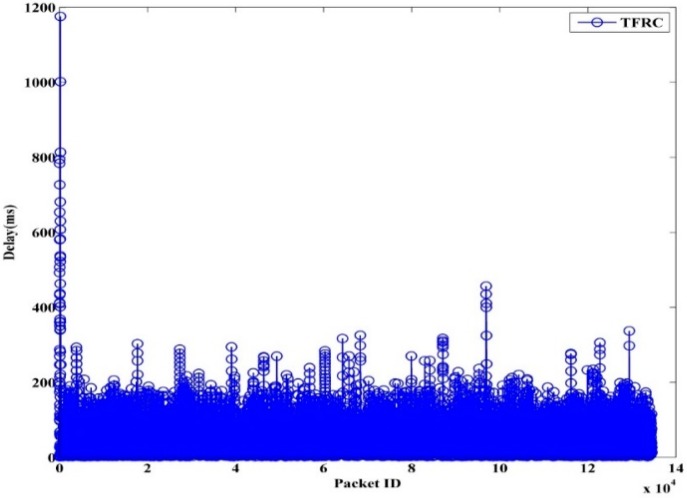
TFRC Packet Delay.

**Figure 14:**
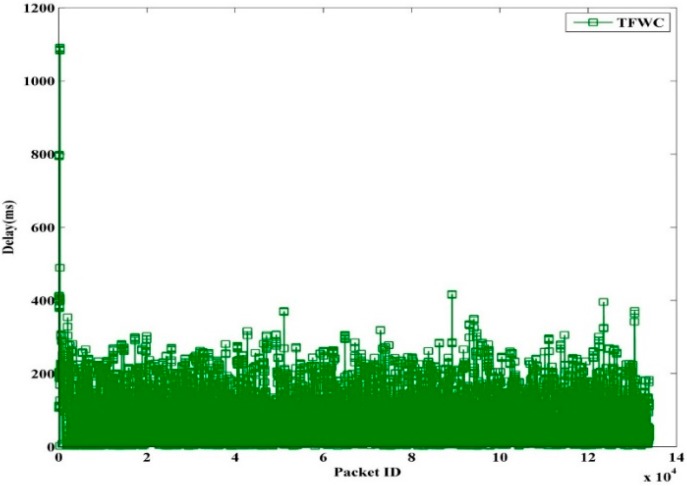
TFWC Packet Delay.

**Figure 15:**
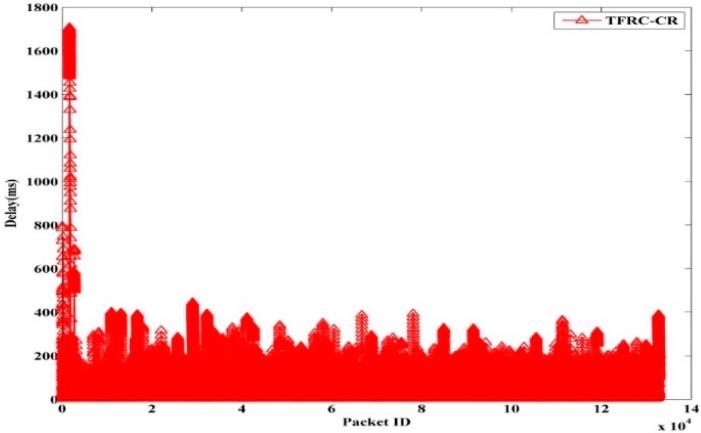
TFRC-CR Packet Delay.

The instantaneous delay of TFRC, TFWC, and TFRC-CR are shown in Figure 13, Figure 14 and Figure 15, respectively. In TFRC, the PU activity forces the source to reduce the transmission rate by half at every time until the minimum rate is reached. As a result, its transmission rate stays low even after the next available channel is used. Therefore, the number of packet transmission is decreased and the delay is reduced. TFWC incurs more delay and higher throughput than TFRC. Initially, it doubles the *cwnd* at each round trip time. On the occurrence of the first packet loss, it halves the *cwnd*. On the occurrence of the successive packet losses, it advances *cwnd* very slowly. However, it is still able to achieve better throughput than TFRC and incurs more delay. TFRC-CR suffers from more delay than TFWC at the cost of more throughput. When the PU is detected, it immediately halts transmission and restarts after the PU becomes inactive. Therefore, it reduces the packet losses, and the transmission rate is less affected due to PU interruption. However, the PU activity causes the queue to build up on the node. Hence, it attains more delay than TFWC and TFRC.

Table 3 shows the average delay for TFRC, TFWC, TFRC-CR and OHTP. It can clearly be seen that our proposed scheme, OHTP, incurs the highest delay. However, that is the tradeoff between high throughput and low packet delay.

**Table 3:** Average Delay (ms).

TFRC	TFWC	TFRC-CR	OHTP
97.62	103.70	114.87	175.75

**Figure 16:**
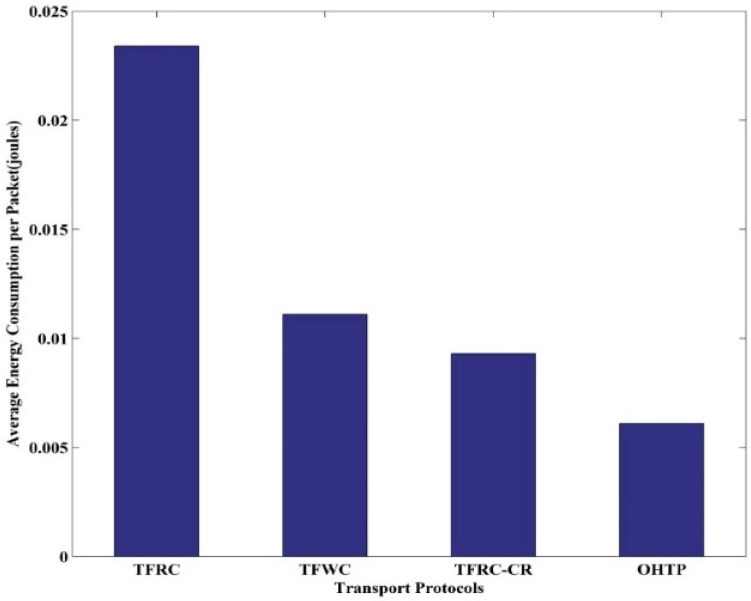
Energy consumption of TFRC, TFWC, TFRC-CR, and OHTP.

In CRASN, nodes dissipate energy in processing, transmitting and receiving messages. Energy consumption is a key aspect in the design of protocols for sensor networks [28]. This energy is needed for correct working of the sensor networks. In our simulation, we used the standard power consumption in each state provided in [29]. Figure 16 shows the average energy consumption per packet for each protocol. It is shown that our proposed protocol consumes the least energy to transmit the packet. Hence, it prolongs the network lifetime.

## 5. Conclusions

The introduction of innovative cognitive radio technologies opens up new research areas to overcome the problem of spectrum scarcity by using it more efficiently. We propose the opportunistic hybrid transport protocol (OHTP) for cognitive radio ad hoc sensor networks to achieve the same goal. We designed and embedded the new congestion control algorithm to handle PU arrival intelligently under the transport protocol. Furthermore, there are pros and cons to window- and rate-based transport protocols. For instance, if the network is greatly congested, and as a result the *cwnd* value remains low, rate-based transmission is well-suited to give smoother throughput. Otherwise, window-based transmission is a better option. Therefore, we propose the hybrid transport protocol. Another issue is to set the optimal threshold value to choose the transmission mode. We empirically calculated and set the threshold value to 70. The results reassure us that we achieved the best outcome. PU interruption detection is also very critical to ensure optimal performance. Helped by this, the transport protocol can differentiate between true and fake congestion. Congestion results in packet losses, and adversely affects the transmission rate of the transport protocol. The phenomenon of packet losses due to PU interruption should not be considered congestion; rather, they are interruption losses. We suggest keeping the same rate, or *cwnd*, for future transmissions to exploit the next available spectrum. This is the opportunistic way to transmit more packets within the given time. In the case of true congestion, we reduce the rate by the same packet loss ratio. The results indicate that our approach is correct. We compared OHTP with TFRC, TFRC-CR, and TFWC. The results show that OHTP is the superlative transport protocol amongst all of them.

## References

[1] Federal Communications Commission Notice of Proposed Rulemaking and Order (FCC 03-222). http://web.cs.ucdavis.edu/~liu/289I/Material/FCC-03-322A1.pdf.

[2] Mitola J. (2000). Cognitive Radio: An Integrated Agent Architecture for Software Defined Radio. Ph.D. Thesis.

[3] Akyildiz I.F., Lee W., Chowdhury K.R. (2009). CRAHNs: Cognitive radio ad hoc networks. Ad Hoc Netw..

[4] Akan O.B., Karli O.B., Ergul O. (2009). Cognitive radio sensor networks. IEEE Netw..

[5] Joshi G.P., Nam S.Y., Kim S.W. (2013). Cognitive radio wireless sensor networks: Applications, challenges and research trends. Sensors.

[6] Postel J. RFC 793—Transmission Control Protocol. https://tools.ietf.org/html/rfc793.

[7] Paxson V., Allman M. RFC 2988—Computing TCP’s Retransmission Timer. https://tools.ietf.org/html/rfc2988.

[8] Fullmer C.L., Garcia-Luna-Aceves J.J. (1997). Solutions to hidden terminal problems in wireless networks. ACM SIGCOMM Comput. Commun. Rev..

[9] Jacobson V. (1995). Congestion avoidance and control. ACM SIGCOMM Comput. Commun. Rev..

[10] Floyd S., Henderson B. RFC 2582—The NewReno Modifications to TCP’s Fast Recovery Algorithm. https://tools.ietf.org/html/rfc6582.

[11] Matins M., Floyd S., Romanow A. RFC 2018—TCP Selective Acknowledgment Options. https://tools.ietf.org/html/rfc2018.

[12] Brakmo L.S., Paterson L.L. (2006). TCP Vegas: End to end congestion avoidance on a global Internet. IEEE J. Sel. Area Commun..

[13] Chowdhury K.R., Felice M.D., Akyildiz I.F. (2013). TCP CRAHN: A transport control protocol for cognitive radio ad hoc networks. IEEE Trans. Mob. Comput..

[14] Tsukamoto K., Koba S., Oie Y. (2015). Cognitive radio-aware transport protocol for mobile ad hoc networks. IEEE Trans. Mob. Comput..

[15] Salim S., Moh S. (2014). A robust and energy-efficient transport protocol for cognitive radio sensor networks. Sensors.

[16] Floyd S., Handley M., Padhye J., Widmer J. Equation-based congestion control for unicast applications. Proceedings of the Conference on Applications, Technologies, Architectures, and Protocols for Computer Communication.

[17] Handley M., Floyd S., Padhye J., Widmer J. RFC 3448—TCP Friendly Rate Control (TFRC): Protocol Specification. https://www.ietf.org/rfc/rfc3448.txt.

[18] Al-Ali A.K., Chowdhury K.R. (2013). TFRC-CR: An equation-based transport protocol for cognitive radio networks. Ad Hoc Netw..

[19] Jourjon G., Lochin E., Senac P. Towards sender-based TFRC. Proceedings of the IEEE International Conference on Communications( ICC).

[20] Renker G., Fairhurst G. IETF Internet Draft draft-renker-dccp-tfrc-rtt-option-01—Sender RTT Estimate Option for DCCP. https://tools.ietf.org/html/draft-renker-dccp-tfrc-rtt-option-01.

[21] Choi S.H., Handley M. Designing TCP-Friendly Window-based Congestion Control for Real-time Multimedia Applications. Proceedings of 7th International Workshop on Protocols for Future, Large-Scale and Diverse Network Transports (PFLDNeT).

[22] Zong X.X., Qin Y., Li L. (2014). Transport protocols in cognitive radio networks: A survey. KSII Trans. Internet Inf. Syst..

[23] Federal Communications Commission Second Memorandum opinion and Order (FCC 10-174). http://transition.fcc.gov/Daily_Releases/Daily_Business/2013/db0417/FCC-13-53-53A1.pdf.

[24] The Network Simulator—NS-2. http://www.isi.edu/nsnam/ns/.

[25] Hespanha J., Bohacek S., Obraczka K., Lee J. Hybrid Modeling of TCP Congestion Control. Proceedings of 4th International Workshop on Hybrid Systems: Computation and Control.

[26] Huang Y., Ghaderi M., Towsley D., Gong W. TCP performance in coded wireless mesh networks. Proceedings of 5th Annual IEEE Communications Society Conference on Sensor, Meshand Ad Hoc Communications and Networks (SECON).

[27] Gamal A.E., Mammen J., Prabhakar B., Shah D. (2006). Optimal throughput-delay scaling in wireless networks—Part I: The fluid model. IEEE Trans. Inf. Theory.

[28] Razzaque M.A., Dobson S. (2014). Energy-efficient sensing in wireless sensor networks using compresses sensing. Sensors.

[29] Feeney L.M., Nilson M. Investigating the energy consumption of wireless network interface in an ad hoc networking environment. Proceedings of 20th Annual Joint Conference of the IEEE Computer and Communications Societies (INFOCOM).

